# Genome duplication improves rice root resistance to salt stress

**DOI:** 10.1186/s12284-014-0015-4

**Published:** 2014-09-02

**Authors:** Yi Tu, Aiming Jiang, Lu Gan, Mokter Hossain, Jinming Zhang, Bo Peng, Yuguo Xiong, Zhaojian Song, Detian Cai, Weifeng Xu, Jianhua Zhang, Yuchi He

**Affiliations:** 1 Hubei Collaborative Innovation Center for Green Transformation of Bio-Resources, Hubei University, Wuhan 430062, P.R. China; 2 School of Life Sciences and State Key Laboratory of Agrobiotechnology, The Chinese University of Hong Kong, Hong Kong, China; 3 State Key Laboratory of Soil and Sustainable Agriculture, Institute of Soil Science, Chinese Academy of Sciences, Nanjing 210008, China; 4 Faculty of Biochemistry and Environmental Engineering, Yunyang Teachers’ College, Shiyan 442000, P.R. China

**Keywords:** Genome duplication, Proton transport, Root, Salt stress, Tetraploid rice

## Abstract

**Background:**

Salinity is a stressful environmental factor that limits the productivity of crop plants, and roots form the major interface between plants and various abiotic stresses. Rice is a salt-sensitive crop and its polyploid shows advantages in terms of stress resistance. The objective of this study was to investigate the effects of genome duplication on rice root resistance to salt stress.

**Results:**

Both diploid rice (HN2026-2x and Nipponbare-2x) and their corresponding tetraploid rice (HN2026-4x and Nipponbare-4x) were cultured in half-strength Murashige and Skoog medium with 150 mM NaCl for 3 and 5 days. Accumulations of proline, soluble sugar, malondialdehyde (MDA), Na^+^ content, H^+^ (proton) flux at root tips, and the microstructure and ultrastructure in rice roots were examined. We found that tetraploid rice showed less root growth inhibition, accumulated higher proline content and lower MDA content, and exhibited a higher frequency of normal epidermal cells than diploid rice. In addition, a protective gap appeared between the cortex and pericycle cells in tetraploid rice. Next, ultrastructural analysis showed that genome duplication improved membrane, organelle, and nuclei stability. Furthermore, Na^+^ in tetraploid rice roots significantly decreased while root tip H^+^ efflux in tetraploid rice significantly increased.

**Conclusions:**

Our results suggest that genome duplication improves root resistance to salt stress, and that enhanced proton transport to the root surface may play a role in reducing Na^+^ entrance into the roots.

## Background

Salt (Na^+^) stress constitutes an important environmental pressure, and elevated Na^+^ levels in agricultural land adversely affects the quantity and quality of crop plants (Buchanan et al. [2005]; Knäblein et al. [2006]; Atkinson and Urwin [2012]; Horie et al. [2012]). Plants must cope with two major stresses under high salinity: osmotic stress (beginning at the early phase under salt stress), which is caused primarily by water deficits in plant tissues, and ionic stress (beginning at the latter phase under salt stress), which can be caused by the accumulation of Na^+^ and Cl^−^ and by disturbance of the K^+^/Na^+^ ratio in plant cells (Yeo and Flowers [1986]; Glenn and Brown [1999]; Blumwald [2000]; Munns and Tester [2008]; Horie et al. [2012]). During evolution, plants have developed several mechanisms to cope with salt stress at the biochemical and molecular level (Zhu [2002]; Shinozaki et al. [2005]; Kronzucker et al. [2006]; Horie et al. [2009]; Hauser et al. [2010]). Among various mechanisms, control of ion movement across tonoplasts (and the plasma membrane) to maintain a low Na^+^ concentration in the cytoplasm is a key cellular factor for survival under salt stress (Baisakh et al. [2012]; Brini and Masmoudi [2012]), and plants are known to maintain low cytoplasmic Na^+^ via intracellular (Fukuda et al. [2004]; Anil et al. [2005]) and extracellular compartmentalization (Anil et al. [2005]). Plants respond to salt stress by restricting the uptake of Na^+^, and adjustment of the cytoplasmic compartment is achieved by producing compatible osmolytes such as proline, mannitol, sorbitol, and glycine betaine ((Greenway and Munns [1980]; Kavi Kishor et al. [2005]; Yamada et al. [2005]; Chyzhykova and Palladina [2006]; Jayasekaran et al. [2006]); Xu and Shi [2007]; (Munns and Tester [2008]; Amirjani [2011]; Chutipaijit et al. [2011])). Ion accumulation in the cytosol (mainly K^+^) and in the vacuole (Na^+^, especially in salt-tolerant cultivars/species) is also important for osmotic adjustment of plant cells ((Gorham et al. [1987]; Gorham et al. [1990]); Glenn and Brown [1999]; (Knäblein et al. [2006])). Osmotic adjustment by solute accumulation inside the cell is essential to reduce the cellular Ψosm against an osmotic gradient between root cells and the outside saline solution, which eventually restores water uptake into roots during salinity stress (Greenway and Munns [1980]).

Roots are important to plants for a wide variety of processes and serve as the major interface between the plant and various biotic and abiotic factors in the soil environment (Smet et al. [2012]). Plants have evolved various strategies and mechanisms to resist salinity stress, in which both anatomical and physiological adaptations play key roles (Lazof and Cheeseman [1986]; Kronzucker et al. [2006]). Solutes and water move radially through the roots via a combination of apoplastic, symplastic, and transcellular pathways. The mechanisms by which Na^+^ enters the shoots of plants remain unclear (Kronzucker and Britto [2011]), but apoplastic transpirational bypass flow of water and solutes is known to play an important role in rice (Yeo et al. [1987]; Ochiai and Matoh [2002]). The apoplastic barriers in roots may also play a major role (Yeo et al. [1987]; Anil et al. [2005]; Gong et al. [2006]). The majority of Na^+^ that enters the shoots of rice plants occurs through “apoplastic bypass,” whereby Na^+^ ions move through the apoplast via solvent drag (Ranathunge et al. [2005]), bypassing Casparian bands (Ochiai and Matoh [2002]; Gong et al. [2006]). In rice, the highly suberized endodermal barrier presents the major resistance to radial water flow (Miyamoto et al. [2001]; Ranathunge et al. [2003]). Casparian bands of the endodermis and exodermis play crucial roles in blocking apoplastic movement of ions and water into the stele of roots through the cortex, and these apoplastic barriers differ considerably in structure and function along the developing root (Chen et al. [2011]; Krishnamurthy et al. [2011]; Zhou et al. [2011]). Characterization of the hydraulic conductivity of roots of both herbaceous and woody species indicated that unfavorable environmental conditions reduce hydraulic conductivity ((Kramer and Boyer [1995]; Steudle and Heydt [1997]; Steudle and Peterson [1998]; Barrowclough et al. [2000]; Miyamoto et al. [2001]; Lee et al. [2005]); Zimmermann et al. [2000]). Furthermore, the chemical composition of suberin in the apoplastic barrier affects the hydraulic conductivity of roots (Schreiber et al. [2005]).

Polyploidy, which is believed to play an important role in plant evolution and breeding, can significantly improve the function of resistance genes and enrich the range of genetic variation in these genes, thus increasing the adaptability of plants to dynamic environments (Adams and Wendel [2005]; Chen and Tian [2007]; Soltis et al. [2009]). The discovery and application of polyploidy meiosis stability (PMeS) material for disrupting a low seed set rate may be used for polyploid rice breeding in the future. Rice plants are very important as food and experimental models (Krishnan et al. [2009]), and polyploid rice may result in evolutionary dominance in terms of stress resistance (Cai et al. [2004]; Cai et al. [2007]; He et al. [2010]; He et al. [2011]). At this time, little information is available regarding the effects of abiotic stress in polyploid rice (Dong and Adams [2011]). As cereal crops, polyploid wheat has been examined under salt stress. In a study on the variation in salt tolerance within a Georgian wheat germplasm collection, the endemic hexaploid winter wheat *Triticum macha* and the endemic tetraploid wheat *Triticum timopheevii* were among the most tolerant to salt stress (Badridze et al. [2009]). In tetraploid wheat genotypes, Na^+^ exclusion correlated well with salinity tolerance in the durum subspecies, and K^+^/Na^+^ discrimination correlated to a lesser degree (Munns and James [2003]). Other studies have shown that salt stress inhibited germination in all wheat genotypes, but the effect was more pronounced in Potohar (hexaploid, salt-sensitive) than other genotypes (Javed, F). Studies on cotton also showed that the subfunctionalization of genes duplicated by polyploidy occurred in response to abiotic stress conditions. Partitioning of duplicate gene expression in response to environmental stress may lead to duplicate gene retention during subsequent evolution (Liu and Adam [2007]). In other plants, some studies have reported that citrus tetraploid genotypes are more tolerant of moderate saline stress than the diploid genotypes, and that citrus tetraploid rootstocks are more tolerant to salt stress than the corresponding diploid rootstock genotypes (Saleh et al. [2008]; Mouhaya et al. [2010]). In hexaploid *Acanthophyllum* species, the negative effects of salinity on some growth parameters, including protein content and antioxidant enzymes, decreased in tetraploid species (Meratanl et al. [2008]). Rice is a salt-sensitive crop considered more sensitive to salt stress during the early seedling than reproductive stage (Flowers and Yeo [1981]; Lutts et al. [1995]; Hasanuzzaman et al. [2009]). Few studies have explored the effect of genome duplication on rice development under salt stress. An earlier study reported that the application of PMeS alleviated the low seed set rate, leading researchers to investigate adaptability under adverse conditions (Cai et al. [2004]; Cai et al. [2007]; He et al. [2010]; He et al. [2011]). Some results suggested that salt stress has a large negative impact on seed germination and seedling growth in rice, but that genome duplication has positive roles in modulating salt stress adjustability in different rice cultivars (Jiang et al. [2013]). Polyploidy is believed to facilitate increased plant adaptability to environmental extremes, and thus characterizing the developmental and morphological changes in roots of polyploid rice that protect against the excessive influx of Na^+^ is important. This may also be promising for screening or generating salt-tolerant polyploid rice varieties. The present study examined the impact of genome duplication on rice roots during saline treatment to increase our understanding on the adaptability of polyploid rice to salt stress and on improving the adaptation of rice under salt stress.

## Results

### The effect of genome duplication on rice root growth under salt stress

**Figure 1:**
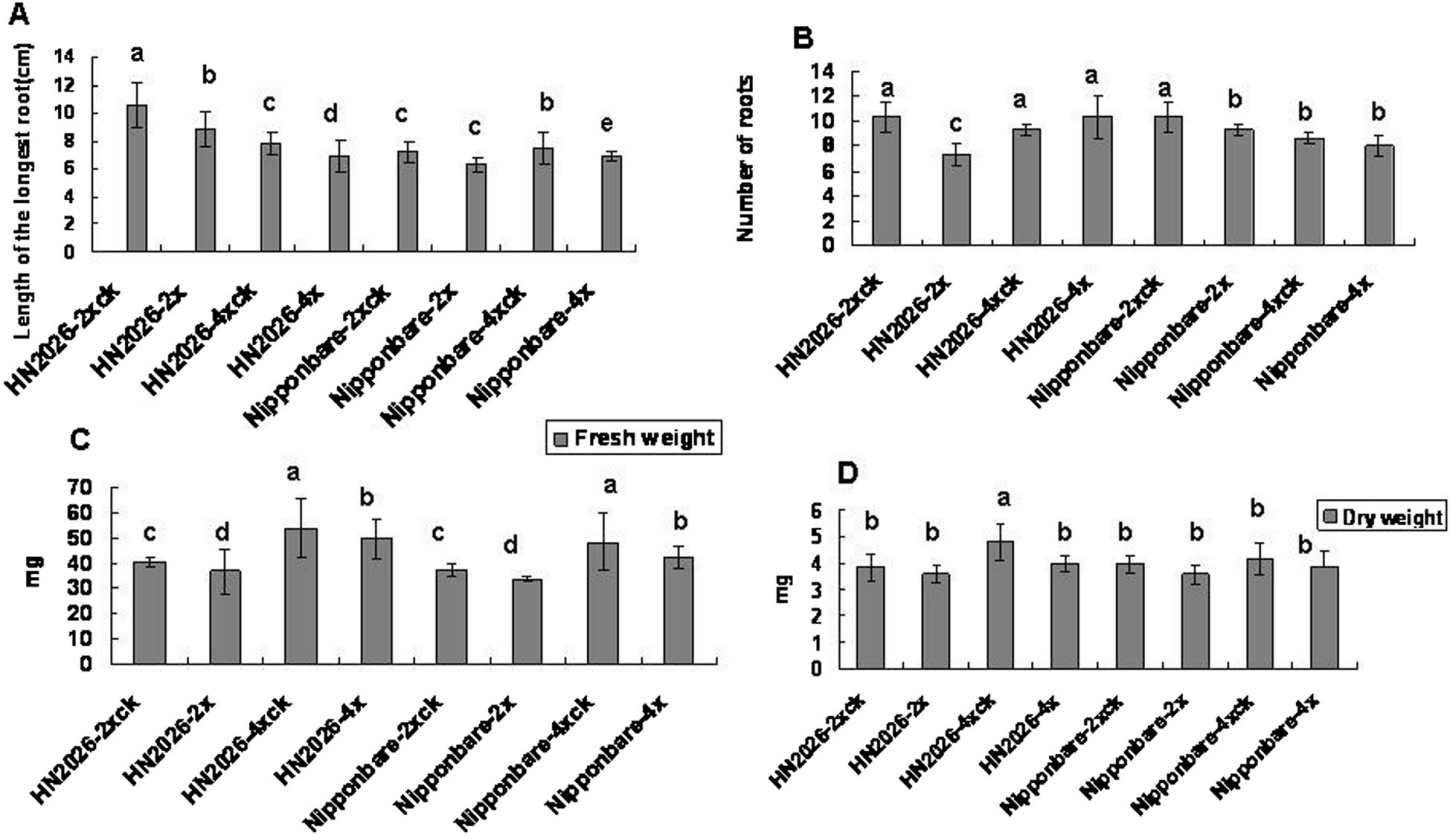
**Root growth in diploid and tetraploid rice cultivars under salt stress for 5 days.** Data represent the mean ± SD (n = 30 × 3 independent biological replicates: all samples were tested in three independent experiments with each included thirty rice plants). Means followed by common letters are not significantly different at P = 0.05 using a protected least-significant difference. Note: **(A)** Length of the longest root; **(B)** The number of roots; **(C)** Fresh weight of the total roots; **(D)** Dry weight of the total roots.

The length, fresh weight, dry weight, and number of roots of polyploid rice cultivars were investigated to characterize the effects of genome duplication under salt stress. Our results demonstrated that salt stress significantly restricted rice root growth, irrespective of being diploid or tetraploid rice, and genome duplication improved root resistance in tetraploid rice by contributing to faster and better root growth in the presence of 150 mM NaCl (Figure 1). Root length was restricted in all species. Salt stress significantly decreased the length of the longest root, irrespective of being diploid or tetraploid rice. Moreover, compared with roots under normal conditions, the restriction degree of root length in the diploid was much stronger than that in the tetraploid. The fresh weight of total roots in tetraploid rice showed a more significant increase than in diploid rice under salt conditions. However, the fresh weight of total roots decreased in all rice tested after salt treatment, and no significant difference in the dry weight of total roots was observed, excluding HN2026-4x. In addition, the results suggested that the total number of roots existed no evident difference in both HN2026-4x and Nipponbare-4x under salt stress, but significant differences were found in the two diploid rice materials with salt treatment (Figure 1).

### Proline accumulation in the roots of diploid and tetraploid rice under salt stress

**Figure 2:**
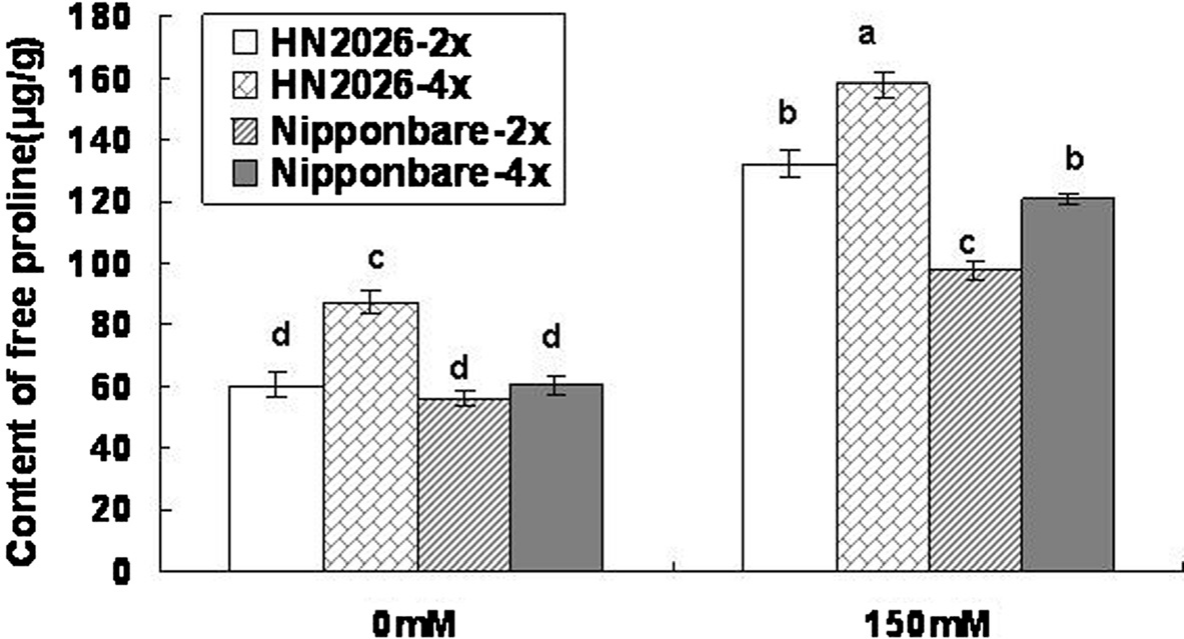
**Amount of free proline in roots of diploid and tetraploid rice under salt stress.** Data represent the mean ± SD (n = 30 × 3 independent biological replicates:all samples were tested in three independent experiments with each included thirty rice plants). Means followed by common letters are not significantly different at P = 0.05 using a protected least-significant difference.

Free proline in roots of diploid and tetraploid rice subjected to 150 mM NaCl for 5 days was measured (Figure 2). The amount of free proline in tetraploid rice cultivars under salt stress varied greatly and increased compared to diploid cultivars. The amounts of free proline in HN2026-2x and Nipponbare-2x were 132.09 and 98.12 μg g^–1^, respectively. Free proline accumulation was highest in HN2026-4x (157.91 μg g^–1^) and reached 120.99 μg g^–1^ in Nipponbare-4x. However, the increase in free proline in Nipponbare-4x compared with Nipponbare-2x was 23.30%. In addition, the increase in HN2026-4x was 19.55%.

### Accumulation of soluble sugar in roots of diploid and tetraploid rice cultivars

**Figure 3:**
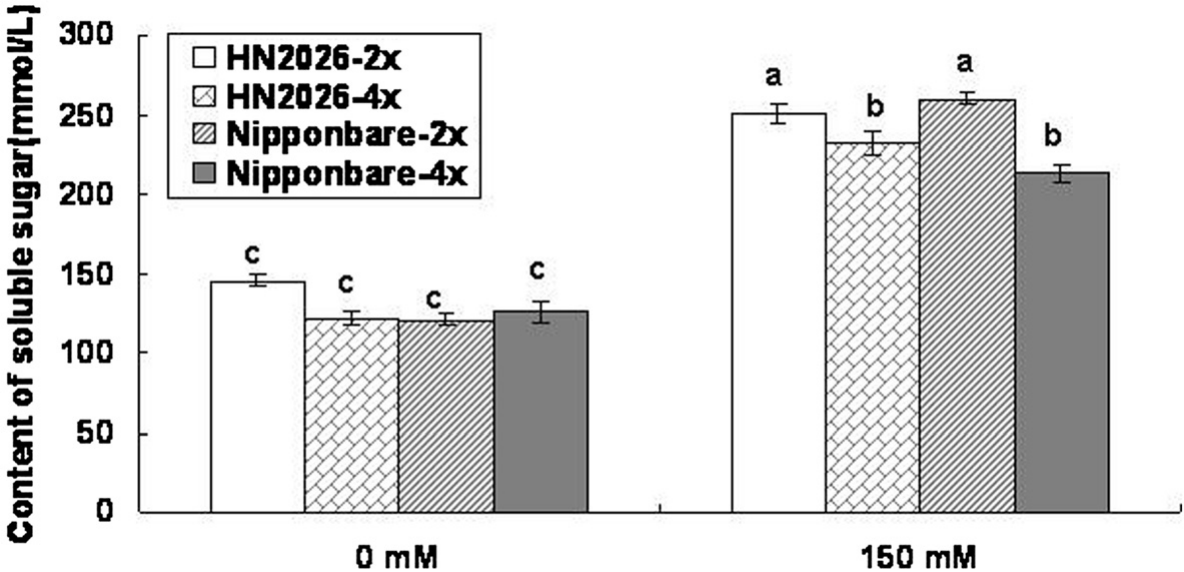
**The amount of soluble sugar in roots of diploid and tetraploid rice under salt stress.** Data represent the mean ± SD (n = 30 × 3 independent biological replicates: all samples were tested in three independent experiments with each included thirty rice plants). Means followed by common letters are not significantly different at P = 0.05 using a protected least-significant difference.

Genome duplication led to a similar increase in different rice cultivars in terms of soluble sugar accumulation under salt stress, and the difference was significant between tetraploid and diploid rice subjected to salt stress. However, no significant changes were found between the two different cultivars for tetraploid or diploid rice (Figure 3). The amount of soluble sugar in tetraploid rice roots was similar to that of the corresponding diploid cultivars under normal conditions, but showed a marked decrease in the tetraploid rice roots compared with diploid cultivars under salt stress.

### The content of malondialdehyde in roots of diploid and tetraploid rice cultivars under salt stress

**Figure 4:**
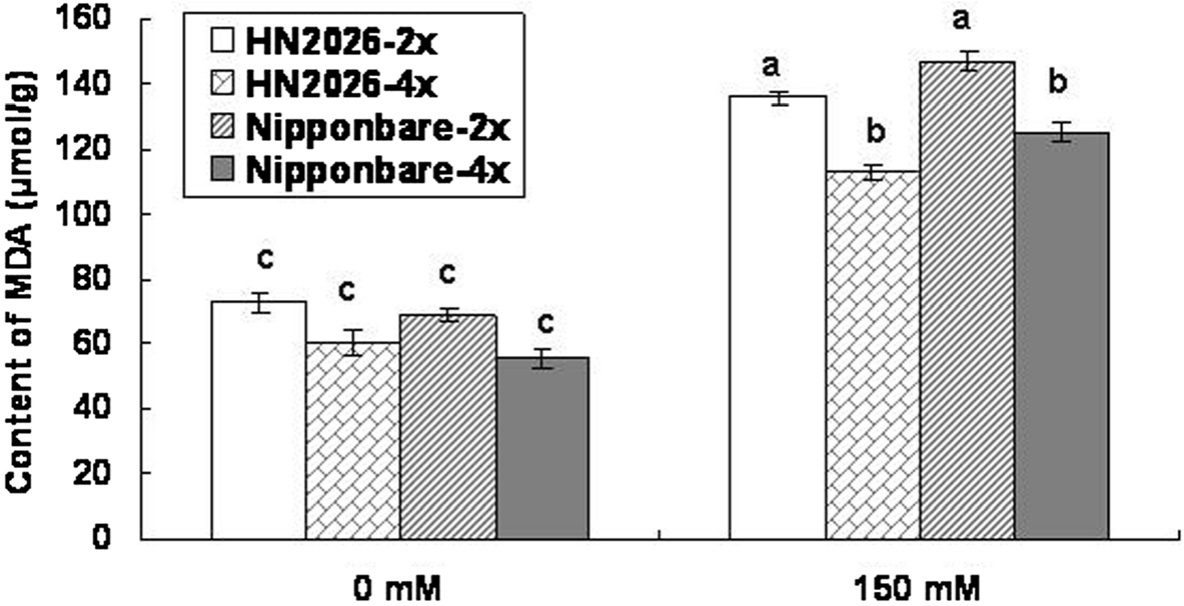
**The accumulation of MDA in roots of diploid and tetraploid rice under salt stress.** Data represent the mean ± SD (n = 30 × 3 independent biological replicates: all samples were tested in three independent experiments with each included thirty rice plants). Means followed by common letters are not significantly different at P = 0.05 using a protected least-significant difference.

Malondialdehyde (MDA) accumulated to similar levels in all rice cultivars tested, and no significant difference was detected between diploid and tetraploid rice cultivars without salt stress. However, the amount of MDA in the roots of various rice cultivars under salt stress was significantly greater than in the control (Figure 4). In contrast, the amount of MDA in the roots of tetraploid rice under salt stress was significantly lower than that in diploid cultivars, suggesting that membrane integrity was higher in tetraploid cultivars than in diploid rice (Figure 4). Genotypes Nipponbare-4x (55.58 μmol g^–1^) and HN2026-4x (60.10 μmol g^–1^) accumulated less MDA in their roots compared to the corresponding diploid cultivars under normal condition. Under salt stress, the amount of MDA in HN2026-4x conditioned with salt was lowest among all cultivars (Figure 4).

### Anatomical structure of roots in diploid and tetraploid rice under salt stress

**Figure 5:**
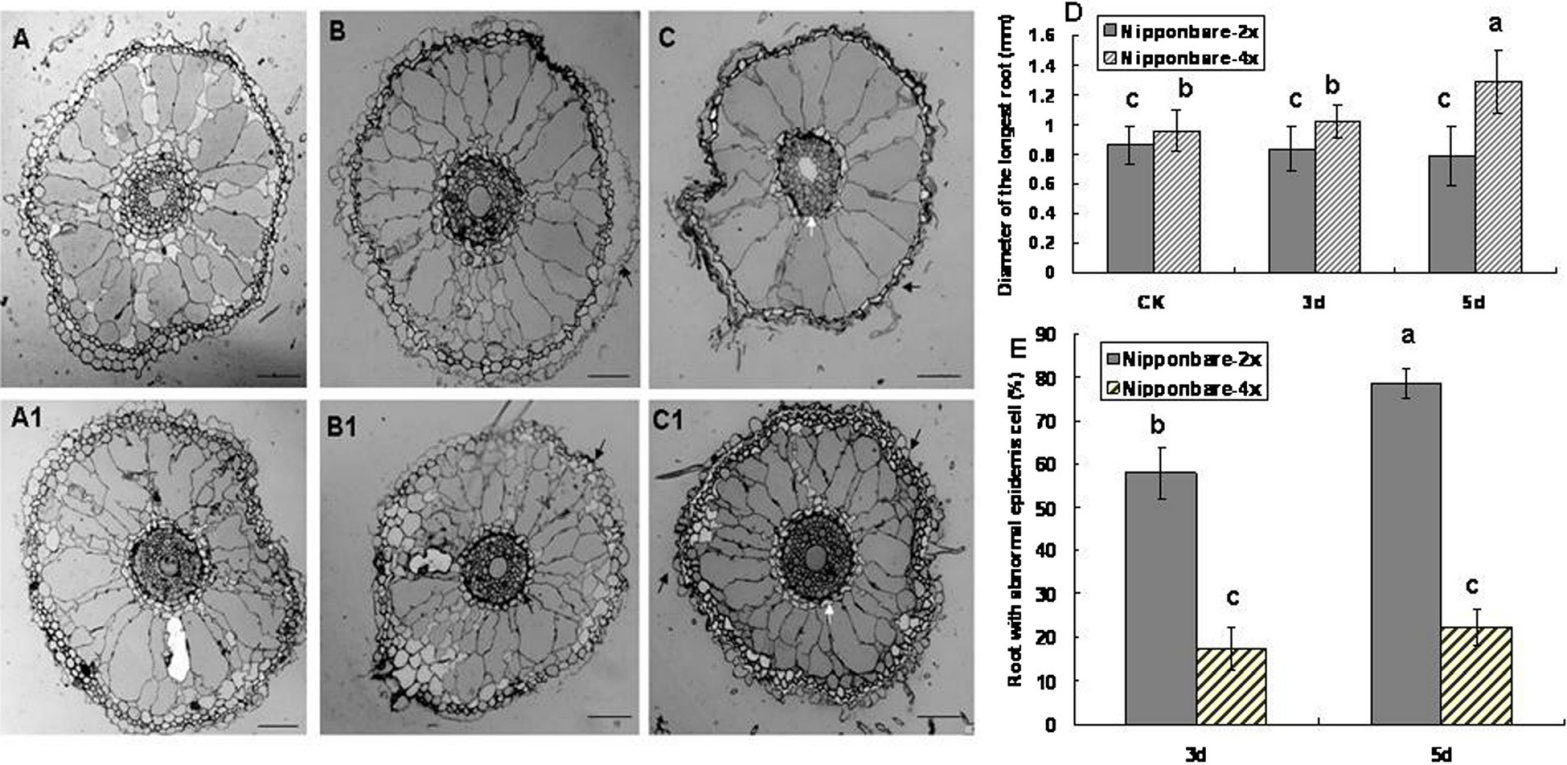
**The longest root microstructure, diameter, and abnormal epidermis frequency of Nipponbare-2x and Nipponbare-4x under salt stress (Bar = 50 μm).(A)** Roots of Nipponbare-2x under normal conditions. **(A1)** Roots of Nipponbare-4x under normal conditions. **(B)** Root of Nipponbare-2x under salt stress for 3 days, whereby the black arrows show the epidermis cells abnormally shelled. **(B1)** Root of Nipponbare-4x under salt stress for 3 days, whereby the epidermis cells maintained a normal station and the black arrow shows regularly thicker endodermis cells. **(C)** Roots of Nipponbare-2x under salt stress for 5 days, whereby the root shrank and transfigured; the black arrow suggests that the epidermis became thinner. **(C1)** Root of Nipponbare-4x under salt stress for 5 days, whereby the protective gap formed between the cortex cells and pericycle cells (white arrow) and the epidermis cells became much thicker (black arrow) and were in close contact with each other. **(D)** Diameter of the longest root; **(E)** Frequency of roots with abnormal epidermis cells under salt stress.

To increase our understanding of the root response in polyploid rice, the anatomical structure of roots in Nipponbare-2x and -4x cultivars were observed on plants under salt stress for 3 and 5 days because Nipponbare-4x was thought to be more resistant to salt. Histological analysis indicated that genome duplication regulated the root response to salt stress. The root microstructure in diploid and tetraploid rice was similar without salt stress, and no evident morphological differences in the epidermis, cortex, vascular system, or aerenchyma were observed to facilitate oxygen transport. However, the diameter of the longest root in tetraploid rice was larger than that in the corresponding diploid (Figure 5A, A1, D). Following 3 days of stress at 150 mM NaCl, evident epidermis cell transfigurations were detected in Nipponbare-2x. For example, it became thinner and 57.89% of roots showed some epidermis cells that were shelled. However, 82.75% of the investigated roots indicated the epidermis cells in Nipponbare-4x maintained the normal framework and became thicker (Figure 5B, B1, E). Continuous morphological analysis after the root was exposed to 150 mM NaCl for 5 days revealed distinct differences between diploid and tetraploid rice (Figure 5C, C1). The roots in Nipponbare-2x shrank and became transfigured because of the extended water absence under salt stress; 78.54% of epidermis cells in the investigated roots brushed off and more aerenchyma tissues were produced by the cortex cells compared to 3-day roots under salt stress(Figure 5E). In addition, the pericycle cells shrank (Figure 5C, E), while in the roots of Nipponbare-4x, only 22.34% of epidermis cells separated from the cortex cell. Obvious thicker epidermis cells were in tight contact, and endo-epidermis formed a thicker barrier protected from the aerenchyma damage and blocked deleterious ion transport to pericycle cells. The protective gap produced between the cortex cells and pericycle cells, as well as the neighboring cells of pericycle cells, were thicker, which was considered the second barrier for the root in Nipponbare-4x. The root response in HN2026-2x and HN2026-4x was similar to Nipponbare-2x and Nipponbare-4x, respectively (data not shown).

### 6 < H2 > Ultrastructural comparison of roots in diploid and tetraploid rice under salt stress

**Figure 6:**
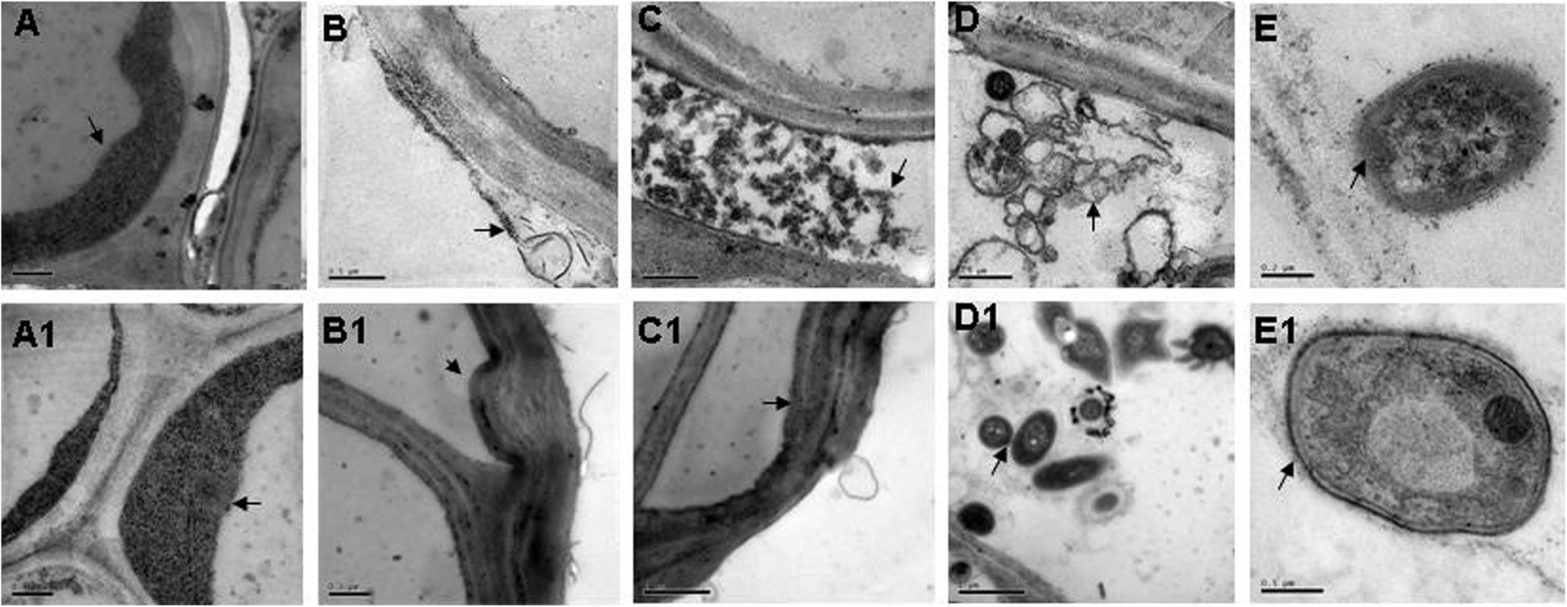
**Root ultrastructure of Nipponbare-2x and Nipponbare-4x under salt stress (Bar in E = 0.2 μm; others bars = 0.5 μm). (A)** The epidermis cells with abundant cytoplasm (arrow) in the diploid without NaCl treatment. **(A1)** The epidermis cells in tetraploid rice were similar to the diploid under normal conditions. Arrow indicates abundant cytoplasm. **(B)** After NaCl treatment for 3 days the cell wall of the epidermis cells became loose (arrow). **(B1)** The cell wall of epidermis cells in Nipponbare-4x became thicker and formed a barrier around the cells (arrow). **(C)** Floccules (arrow) were discovered between the epidermis cells in Nipponbare-2x. **(C1)** The epidermis cells were normal in Nipponbare-4x, and the arrow shows normal abundant cytoplasm. **(D)** Membrane organelles were indicative of evident transfiguration (arrow) in the pericycle cells of Nipponbare-2x. **(D1)** Pericycle cells maintained a normal shape surrounded by cytoplasm (arrow) in Nipponbare-4x. **(E)** The nuclei with an abnormal shape, whereby floccule nuclear cytoplasm was observed in the pericycle cells of Nipponbare-2x (arrow). **(E1)** Nuclei with intact membrane (arrow) and dispersed chromatins were observed in pericycle cells of Nipponbare-4x.

Ultrastructural analysis in Nipponbare-2x and -4x cultivars roots under 150 mM NaCl stress for 3 and 5 days showed that genome duplication improved rice adaptability, including the epidermis cell protective mechanism, membrane organelle, and nuclei stability. The epidermis cells with abundant cytoplasm accumulation around the cell wall were closely connected in diploid and tetraploid rice without salt treatment (Figure 6A, A1). After treatment with NaCl for 3 days, the cell wall of the epidermis cells became loose, and some exterior parts of the epidermis cell wall were isolated and desquamated in Nipponbare-2x (Figure 6B). However, in Nipponbare-4x, the epidermis cell wall became thicker and a barrier formed around the epidermis cells (Figure 6B, B1). A significant difference was observed for the cortex cells between Nipponbare-2x and Nipponbare-4x. Floccules were discovered between the cortex cells in Nipponbare-2x (Figure 6C), which was not observed in Nipponbare-4x (Figure 6Cl). Membrane organelles showed an evident transfiguration in the pericycle cells of Nipponbare-2x (Figure 6D). In contrast, cells maintained their normal shape surrounded by cytoplasm in Nipponbare-4x (Figure 6D1). After NaCl treatment for 5 days, nuclei with an abnormal shape and floccule nuclear cytoplasm were observed in the pericycle cells of Nipponbare-2x (Figure 6E). However, nuclei with intact membranes, smooth surfaces, and dispersed chromatin were observed in Nipponbare-4x (Figure 6E1). These results indicated that genome duplication promoted roots to show normal active metabolism.

### Na^+^ content and H^+^ flux in diploid and tetraploid rice under salt stress

**Figure 7:**
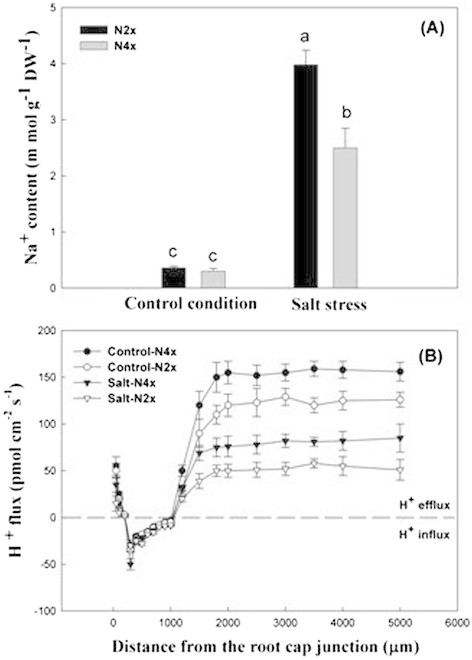
**Na**^**+**^**content and H**^**+**^**flux of Nipponbare-2x and -4x under salt stress. (A)** Na^+^ content in Nipponbare-2x and -4x. **(B)** H^+^ flux in Nipponbare-2x and -4x.

The Na^+^ content in the whole plant including the root and shoot was measured using inductively coupled plasma emission spectroscopy (ICP-AES)( Figure 7A). The results clearly indicated that Na^+^ accumulation in Nipponbare-2x and Nipponbare-4x did not differ from control conditions, whereas Na^+^ content in Nipponbare-2x increased significantly compared to Nipponbare-4x under salt stress. This low level of Na^+^ content suggested that Nipponbare-4x had a better protective effect against deleterious ions, leading to higher salt tolerance. H^+^ efflux and influx were detected in the roots of Nipponbare-2x and Nipponbare-4x, which was demonstrated 500 μm from the root tip (Figure 7B). We observed that H^+^ efflux or influx in Nipponbare-2x and Nipponbare-4x did not differ between control and treated conditions. At 500–1000 μm from the root tip, H^+^ influx was dominant, which was similar in diploid and tetraploid rice. However, H^+^ efflux increased gradually as distance increased to 1000–2000 μm from the root tip, which was higher in Nipponbare-4x than in Nipponbare-2x under both control conditions and salt stress. Subsequently, H^+^ efflux entered into the stable stage beyond 2000 μm from the root tip, and a striking difference was observed between H^+^ efflux in Nipponbare-4x and Nipponbare-2x under salt treatment. The high H^+^ efflux was indicative of low pH in Nipponbare-4x.

## Discussion

Salinity is an important environmental factor limiting the productivity of crop plants because most crop plants are sensitive to high concentrations of Na^+^ in the soil (Munns and Tester [2008]; Ahmad and Prasad [2012]; Hasanuzzaman et al. [2013]). Salinity at higher levels caused both hyperionic and hyperosmotic stress, and resulted in a series of adverse effects that ultimately resulted in plant death (Mahajan and Tuteja [2005]; Hasanuzzaman et al. [2012]; Brini and Masmoudi [2012]). As we know, osmotic stress was beginning at the early phase under salt stress, and then ionic stress was started at the latter stress phase (Greenway and Munns [1980]; Al-Khayri and Al-Bahrany [2002]; Yamada et al. [2005]; Jayasekaran et al. [2006]; Munns and Tester [2008]; Krishnamurthy et al. [2009]; Szabados and Savouré [2010]).The rice roots, which are in direct contact with the soil, must tolerate osmotic and ionic stress under saline conditions (Smet et al. [2012]). Osmotic stress in plants occurs immediately with an increase in salt levels outside the roots, which inhibits water uptake, cell expansion, and lateral bud development (Steudle [2000]; Munns [2002]; Ranathunge et al. [2003]; Ranathunge et al. [2005]; Munns and Tester [2008]). On the other hand, salt-induced oxidative stress may disrupt the membrane structure since the overproduction of reactive oxygen species triggers lipid and protein peroxidation ((Dionisio-Sese and Tobita [2000]); Radic et al. [2006]; (Moller et al. [2007]; Ahmad et al. [2009]; Azevedo et al. [2009])). Our results indicated that the amount of free proline in the roots of tetraploid rice was siginificantly higher than that in corresponding diploid cultivars, however, the amount of MDA in roots of all tetraploid rice cultivars was markedly less than that in the diploid cultivars, which suggests that tetraploid rice can maintain membrane integrity under salt treatment .Thus, we proposed that the differences among proline and MDA between tetraploid rice and diploid rice may contribute to salt-tolerance in early-salt-stress response and help to combat with later-salt-stress response in rice.

The ionic stress phase develops later when toxic ions such as Na^+^ accumulate in plants, particularly when transport from root to leaves is over the threshold ((Yeo and Flowers [1986]); Glenn and Brown [1999]; Zhu [2002]). Roots are thought to cope with ionic stress during high salt treatment by adapting their structures, and determining how roots avoid the influx of excess salt is important. Na^+^ translocation from the root to the shoot is an important issue in salt-stress physiology ((Flowers et al. [1977]); Läuchli [1984]; (Tester and Davenport [2003]; Krishnamurthy et al. [2009]; Krishnamurthy et al. [2011])). Based on earlier physiological and morphological studies, the initial uptake of solutes is generally believed to occur at the epidermis or exodermis, or if soil solution flows apoplastically across the root cortex, the endodermis (Enstone et al. [2003]). In most plants, radial transport of Na^+^ into the root xylem occurs through a cell-to-cell pathway involving xylem loading transporters (Munns [2002]). However, in rice, considerable apoplastic bypass flow of Na^+^ into the stele has been observed (Yeo et al. [1987]; Yadav et al. [1996]; Garcia et al. [1997]; Gong et al. [2006]), which is regulated by Ca^2+^ to varying degrees among different rice cultivars (Anil et al. [2005]). Casparian bands of the endodermis and exodermis play crucial roles in blocking apoplastic movement of ions and water into the stele of roots through the cortex (Steudle and Peterson [1998]; Schreiber et al. [1999]; Schreiber et al. [2005]). In this study, to explore the detailed resistance mechanisms to salinity stress in rice roots, the anatomical structure and ultrastructure of roots in Nipponbare-2x and -4x were investigated in plants under salt stress. Our results suggest that epidermis cells in Nipponbare-4x maintained the normal framework after 3 days of salt treatment and showed thicker cell walls of some cortex cells, resulting in the formation of a barrier block near the epidermis. However, in the epidermis of Nipponbare-2x, a series of abnormal changes (such as becoming thinner and cellular distortion) were observed. Continuous root morphological analysis after 150 mM NaCl treatment for 5 days suggested that epidermis cells in Nipponbare-4x became thicker as the first protective barrier against Na^+^. As a protective gap between the cortex cells and pericycle cells, it may be the second protective barrier for the root of tetraploid rice under salt stress. Based on subsequent ultrastructure detection, membrane organelles maintained their normal shape surrounded by cytoplasm under the high salt treatment in Nipponbare-4x. This hypothesis agreed with other previous results, which indicated that excess salts adversely affect all major metabolic activities in rice including cell wall damage, accumulation of electron-dense proteinaceous particles, plasmolysis, cytoplasmic lysis, and endoplasmic reticulum (ER) damage (Steudle and Heydt [1997]; Barrowclough et al. [2000]; Miyamoto et al. [2001]; Lee et al. [2005]). However, our results confirmed that Na^+^ content in Nipponbare-2x significantly increased compared with that in Nipponbare-4x under salt stress. The low level of Na^+^ content suggested that Nipponbare-4x experienced a faint absorption of deleterious ions. We speculated that the anatomical structure and ultrastructure of roots in Nipponbare-4x may play critical roles in counteracting ionic stress at the latter phase under salt stress. Polyploidy can alter plant morphology, phenology, and physiology, increasing plant tolerance to fluctuating environments (Adams and Wendel [2005]). We next explored which factor resulted in Nipponbare-4x salt stress resistance by characterizing the cellular and molecular mechanisms. Several categories of regulatory function and transporter activity were over-duplicated, and the complexity of regulatory networks and adaptability to changing environmental conditions would be increased in polyploidy (Osborn et al. [2003]; Blanc and Wolfe [2004]; Saleh et al. [2008]). Adaptation to stress by high salt content may also occur through gene duplication, and polyploidy has been associated with resistance to high salt concentrations in *Citrus* and *Sorghum*. Polyploidy has been suggested to be a general physiological adaptive response to osmotic stress ((Ceccarelli et al. [2006]; Gerstein et al. [2006]; Saleh et al. [2008]); Dhar et al. [2011]). Polyploidy was advantageous because of the increased vigor compared with diploid and tetraploid relatives and its ability to produce diverse gene products under stress environments (Comai [2005]). However, how polyploid rice increases salt stress tolerance is complicated and requires further study.

Furthermore, under salt stress, the pumping activity of the plasmalemma H^+^-ATPase is inhibited and may contribute to a weaker acidification of the apoplast, and thus to growth inhibition (Maeshima [2001]; (Mariko et al. [2009]; Xu et al. [2013])). Na^+^ sequestration into the vacuole depends on the expression and activity of the Na^+^/H^+^ antiporter that is driven by an electrochemical gradient of protons generated by the vacuolar H^+^-ATPase and H^+^-pyrophosphatase (Fuglsang et al. [2011]; Brini and Masmoudi [2012]). The cell wall extensibility is reduced under salt stress due to the inhibition of pumping activity of the plasmalemma H^+^-ATPase (Zörb et al. [2005]). At a 2000 μm distance from the root cap, H^+^ efflux becomes the dominant form with stable values, and Nipponbare-4x was significantly higher than Nipponbare-2x under salt treatment. The plasmalemma-H^+^-ATPase acidifies the apoplast by pumping protons out of the cell, and the decreased pH activates cell wall-loosening enzymes that break bonds in the cell wall and enables turgor to drive cell elongation (Michelet and Boutry [1995]; Palmgren [1998]; Gaxiola et al. [2002]; Cosgrove [2005]; Gaxiola et al. [2007]). The high H^+^ efflux in tetraploid rice leading to low pH conditions may contribute to faster root growth, but the length, fresh weight, and dry weight of the root were restricted in diploid cultivars compared to tetraploid rice under salt stress.

Salt stress induces various complex biochemical, molecular, cellular, and physiological changes in plants (Atkinson and Urwin [2012]; Horie et al. [2012]). Previous studies have shown that abiotic stress conditions have considerable effects on duplicate gene expression in polyploids, with the effects varying in relation to gene, stress, and organ (Blanc and Wolfe [2004]). Differential expression in response to environmental stress may play a role in the preservation of some duplicated genes in polyploidy (Blanc and Wolfe [2004]). Recent molecular, physiological, and molecular genetic studies have increased our understanding on the protection mechanisms that plants use to cope with detrimental effects of salinity stress ((Blumwald [2000]); Zhu [2002]; (Munns [2005]; Ren et al. [2005]; Munns and Tester [2008]; Horie et al. [2009]); Hauser et al. [2010]). Correlations between the ploidy levels and morphological traits in wheat were significantly positive under saline conditions, showing that values of morphological traits increased with the number and types of genomes. Polyploidy was significantly associated with the species performance for all traits in the study, excluding the number of yellow leaves and shoots under saline conditions (Rauf et al. [2010]). The expression of genes duplicated by polyploidy (termed homeologs) in cotton can be partitioned between the duplicates so that one copy is expressed and functions only in some organs, and the other copy is expressed only in other organs, indicative of subfunctionalization. These results suggest that the subfunctionalization of genes duplicated by polyploidy occurred in response to abiotic stress conditions. Partitioning of duplicate gene expression in response to environmental stress may lead to duplicate gene retention during subsequent evolution (Liu and Adam [2007]). As several sources of improved Na^+^ “exclusion” are now known to reside on different chromosomes in various genomes of species in the Triticeae, further studies are required to identify the underlying mechanisms controlling genes for the various traits that could act additively or even synergistically, which may enable substantial gains in salt tolerance (Colmer et al. [2006]). The regulation mechanism is complicated in polyploid rice, and understanding how duplicated genes affect rice development under salt stress could be important for biological and agricultural applications. The results of our work suggest that tetraploid rice has a better protective mechanism than diploid rice against salinity, in agreement with the results of earlier studies on the genome duplication effect in *Citrus*, *Acanthophyllum* spp., and other plants under salt stress (Saleh et al. [2008]; Yildiz and Terzi [2008]; Mouhaya et al. [2010]). Several studies have indicated that the response of plant cells to high salt is controlled by multiple genes (Bartels and Sunkar [2005]; Chinnusamy et al. [2005]; Sahi et al. [2006]). Polyploid rice was believed to improve the root adaptability to salt stress by regulating root growth and protective structure formation, subsequently decreasing Na^+^ assimilation. Thus, exploring the significance of protection mechanisms in polyploid rice salt tolerance, including morphological barriers at the molecular, cellular, and whole plant level, is important to develop high-yielding, salt-tolerant polyploid cultivars.

## Conclusions

Rice is a very important and salt-sensitive crop. Previous reports have suggested that polyploid rice has some superiority in stress resistance. However, few studies have focused on the effect of genome duplication on rice root response under salt stress. The objective of this study was to investigate how genome duplication regulates the rice root response to salt stress. Our results demonstrated that salt stress significantly restricted rice root growth in both diploid and tetraploid rice, and that genome duplication improved the root growth in tetraploid rice, with faster and better root growth in the presence of 150 mM NaCl. Free proline accumulated in tetraploid rice cultivars under salt stress varied greatly, which increased compared to that in the diploid cultivars. Genome duplication significantly decreased the MDA content in tetraploid rice compared to diploid cultivars subjected to salt stress, which suggests that the membrane integrity improved in the tetraploid compared to the diploid. Investigation of the anatomical structure of roots under salt stress showed a high frequency of epidermis cells maintaining their normal structure, and a gap appeared between the cortex and pericycle cells in tetraploid rice roots. These protective mechanisms improved the root adaptability to salt stress. Ultrastructural analysis showed that genome duplication also improved the root response, including the epidermis cell protective mechanism formation, and membrane organelle and nuclei stability. Anatomical structure and ultrastructure of roots in Nipponbare-4x may play critical roles in counteracting Na^+^ absorption, and Na^+^ content in Nipponbare-2x greatly increased compared to that in Nipponbare-4x under salt stress. The high H^+^ efflux in tetraploid rice led to low pH conditions and may have contributed to increased root growth, the length of the root, and the fresh and dry weights of the root, which were restricted in the diploid compared to the tetraploid under salt stress. Overall, our results suggest that genome duplication improved root resistance to salt stress and enhanced proton transport to the root surface, which may play a role in reducing Na^+^ entry into the roots.

## Methods

### Plant materials and growth conditions

HN2026-2x and -4x and Nipponbare-2x and -4x were used in this study. The tetraploid rice cultivars were cultured according to our patent and as described previously (Cai et al. [2004]). Seeds were germinated on moist tissue paper at 28°C in the dark for 2–3 days. Seedlings were transferred to half-strength Murashige and Skoog (½ MS) medium (Murashige and Skoog [1962]) in containers. The seedlings were allowed to grow for 20 days post-germination with continuous media aeration at 28°C illuminated at 450 μmol m^–2^ s^-–1^ using fluorescent lighting with a day and night cycle of 12 h each. Seedlings were then cultured in ½ MS medium (Murashige and Skoog [1962]) with 150 mM NaCl.

### Measument of root growth under salt stress

We choosed the longest root (primary root as noted in (Xu et al. [2013])) of every plant to measure the length, and weighed the fresh and dry weight of total root with precision electronic autobalance, and recorded the total root numbers. The diameter of the longest root was measured by vernier caliper. All samples were tested in three independent experiments with each included thirty rice plants.

### Extraction of free proline

The proline content in roots in the presence of 150 mM NaCl for 5 days was investigated. The free proline content was extracted and quantified using the acid ninhydrin method as described by Bates et al. (Bates et al. [1973]). The content of free proline was calculated using the standard curve. All samples were tested in three independent experiments with three replicates each.

### MDA content

The content of MDA in roots subjected to 150 mM NaCl for 5 days was calculated. The method was in accordance with the results of Hodges et al. (Hodges et al. [1999]). Briefly, 0.5 g of fresh rice roots was homogenized with 5 ml of 5% (v/v) 2,4,6-trichloroanisole, and then centrifuged at 3000 rpm for 10 min. The supernatant was mixed with 2 ml of 0.67% (v/v) 2,4,6-tribromoanisole, boiled for 30 min, and then cooled and centrifuged again. The absorbance (A) of the supernatant at 532 nm, 600 nm, and 450 nm was measured and the MDA content (C) was calculated as follows: C (μmol L^–1^) = 6.45 (A_532_–A_600_) – 0.56 A_450_. All samples were tested in three independent experiments with three replicates each.

### Soluble reducing sugar content

The soluble reducing sugar content in roots exposed to salt treatment for 5 days was measured as described previously (Ranney et al. [1991]). Briefly, 0.1 g of fresh rice roots was homogenized with 5 ml of distilled water and then centrifuged at 8000 rpm for 10 min. The soluble reducing sugar content was measured as follows: 1.0 ml supernatant and 0.5 ml 3,5-dinitrosalicylic acid were mixed, heated in boiling water for 5 min, and cooled. The absorbance was measured at 520 nm, and the amount of reducing sugar was calculated from the standard curve. All samples were tested in three independent experiments with three replicates each.

### Anatomical analysis of roots

Rice roots exposed to salt treatment for 3 and 5 days were dissected and vacuum-infiltrated with 3% (w/v) paraformaldehyde (Sigma, St. Louis, MO, USA) and 0.25% glutaraldehyde (Sigma) in phosphate-buffered saline (PBS) for 30 min (pH 7.2). The fixed roots were renewed with fresh solution and post-fixed in 1% OsO_4_ (Sigma) in PBS (pH 7.2). The tissues were washed in PBS, dehydrated in a graded ethanol series, and embedded in EPON812 (Emicron, http://www.instrument.com.cn/). Half-thin sections (100 nm) were examined at every stage, and observations and photographic recordings were performed with a BX51 microscope (Olympus, Tokyo, Japan). Ultrathin sections (50–70 nm) were double-stained with 2% (w/v) uranyl acetate (Sigma) and 2.6% (w/v) lead citrate (Sigma) aqueous solution and examined with a transmission electron microscope (H-8100; Hitachi, Tokyo, Japan) at 100 kV.

### Measurement of Na^+^ concentration

After seedlings were cultured in ½ MS medium (Murashige and Skoog [1962]) with 150 mM NaCl for 5 days, whole plants including shoots and roots were collected and dried at 70°C for at least 3 days, after which they were weighed. Samples were digested with HNO_3_ and the concentration of Na^+^ was determined using ICP-AES (IRIS Advantage; Thermo Electron, Waltham, MA, USA). All samples were tested in three independent experiments with three replicates each.

### Assay of H^+^ flux in the rice root tip

H^+^ fluxes were measured noninvasively using SIET (SIET system BIO-003A; YoungerUSA Science and Technology Corporation, Amherst, MA, USA). Rice plants were equilibrated in measuring solution for 20–30 min, and these equilibrated rice plants were transferred to the measuring chamber, which was a small plastic dish (3 cm diameter) containing 2–3 ml of fresh measuring solution. When the root became immobilized at the bottom of the dish, the microelectrode was vibrated in the measuring solution between two positions (5 μm and 35 μm from the root surface) along an axis perpendicular to the root. The background was recorded based by vibrating the electrode in measuring solution not containing roots. The microelectrode was made and silanized by Xuyue Science and Technology Co., Ltd. (Beijing, China). All samples were tested in three independent experiments with three replicates each.

### Statistical analysis

All values are shown as the mean of five replicates, and the average was calculated. The results were analyzed for variance using the SAS/STAT statistical analysis package (version 6.12; SAS Institute, Cary, NC, USA) to determine significant differences. Means followed by common letters are not significantly different at P = 0.05 using a protected least-significant difference.

## Competing interests

The authors declare that they have no competing interests.

## Authors’ contributions

Yi Tu and Aiming Jiang contribute equally to this paper, they cooperted to finish all experiments. Lu Gan, Md. Mokter Hossain and Jinming Zhang made contribution of making figure and table. Bo Peng, Yuguo Xiong and Zhaojian Song were responsible for materails planting , nursing and data analysis. Detian Cai and Jianhua Zhang gave this research important guidance and revised the manuscript. Yuchi He and Weifeng Xu cooperated to design the the whole research and write the manuscript, they are sharing the corresponding person for giving final approval of the version to be submitted. All authors read and approved the final manuscript.
